# Immunohistochemical Analysis of the Natural Killer Cell Cytotoxicity Pathway in Human Abdominal Aortic Aneurysms

**DOI:** 10.3390/ijms160511196

**Published:** 2015-05-18

**Authors:** Irene Hinterseher, Charles M. Schworer, John H. Lillvis, Elizabeth Stahl, Robert Erdman, Zoran Gatalica, Gerard Tromp, Helena Kuivaniemi

**Affiliations:** 1 Department of General, Visceral, Vascular and Thoracic Surgery, Charité Universitätsmedizin, Campus Mitte, 10117 Berlin, Germany; E-Mail: irene.hinterseher@charite.de; 2 Sigfried and Janet Weis Center for Research, Geisinger Health System, Danville, PA 17822, USA; E-Mails: cmschworer@gmail.com (C.M.S.); ecs40@pitt.edu (E.S.); rerdman@geisinger.edu (R.E.); gctromp@geisinger.edu (G.T.); 3 The Center for Molecular Medicine and Genetics, Wayne State University School of Medicine, Detroit, MI 48202, USA; E-Mail: johnlillvis@gmail.com; 4 Caris Life Sciences, Phoenix, AZ 85040, USA; E-Mail: zgatalica@carisls.com; 5 Department of Surgery, Temple University School of Medicine, Philadelphia, PA 19140, USA

**Keywords:** human aorta, immunohistochemistry, double-staining, AAA, aortic aneurysm

## Abstract

Our previous analysis using genome-wide microarray expression data revealed extreme overrepresentation of immune related genes belonging the Natural Killer (NK) Cell Mediated Cytotoxicity pathway (hsa04650) in human abdominal aortic aneurysm (AAA). We followed up the microarray studies by immunohistochemical analyses using antibodies against nine members of the NK pathway (VAV1, VAV3, PLCG1, PLCG2, HCST, TYROBP, PTK2B, TNFA, and GZMB) and aortic tissue samples from AAA repair operations (*n* = 6) and control aortae (*n* = 8) from age-, sex- and ethnicity-matched donors from autopsies. The results confirmed the microarray results. Two different members of the NK pathway, HCST and GRZB, which act at different steps in the NK-pathway, were actively transcribed and translated into proteins in the same cells in the AAA tissue demonstrated by double staining. Furthermore, double staining with antibodies against CD68 or CD8 together with HCST, TYROBP, PTK2B or PLCG2 revealed that CD68 and CD8 positive cells expressed proteins of the NK-pathway but were not the only inflammatory cells involved in the NK-pathway in the AAA tissue. The results provide strong evidence that the NK Cell Mediated Cytotoxicity Pathway is activated in human AAA and valuable insight for future studies to dissect the pathogenesis of human AAA.

## 1. Introduction

Abdominal aortic aneurysm (AAA) is a complex disease of the aging population that is associated with significant morbidity and mortality [1,2,3]. Aneurysms form through a complex remodeling process of the vessel wall involving inflammatory infiltration, degradation of extracellular matrix (ECM) and the loss of smooth muscle cells (SMC), all of which contribute to weakening and dilatation of the aorta [4]. Since the initial characterization of lymphocytes and macrophages in excised AAA tissue [5,6,7] infiltrating immune cells have been increasingly recognized as critical mediators in the pathogenesis of AAA through a variety of functions including release of proinflammatory cytokines, secretion of ECM degrading proteases, and production of reactive oxygen species [3,4,8].

**Figure 1:**
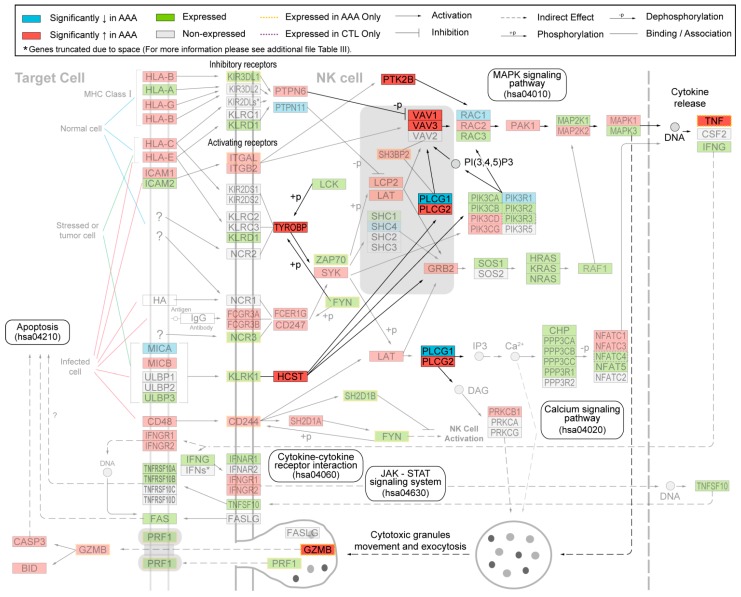
Modified “Natural Killer Cell Mediated Cytotoxicity” (hsa04650) pathway from Kyoto Encyclopedia of Genes and Genomes (KEGG). Protein symbols were replaced by gene symbols. Proteins investigated in the current study using immunohistochemical analysis of human aortic tissue samples from abdominal aortic aneurysm (AAA) and non-AAA samples are shown in bright colors. See key for explanation of colors and symbols. The figure is modified from our previous study [10].

Whole genome expression profiling is an unbiased method to identify the molecular pathways active in AAA tissue by providing a global snapshot of differentially expressed genes [9]. In the only whole genome microarray study comparing AAA tissue with age-matched controls, Lenk *et al.* found a significant upregulation of genes and pathways related to immune response and inflammation [10], including genes previously identified in AAA tissue such as *MMP9*, *IL1* and *CTSH*. Based upon pathway analysis using functional annotation from the Kyoto Encyclopedia of Genes and Genomes (KEGG) [11], the most significantly upregulated set of genes was the KEGG pathway “Natural Killer (NK) Cell Mediated Cytotoxicity” (Figure 1). Each KEGG pathway is a collection of genes with well-described interactions; the genes in the “NK Cell Mediated Cytotoxicity” pathway collectively form a set of signaling pathways well-described in the activation, inhibition and down-stream actions of NK cells. The activation of the NK pathway is initiated by contact of the NK cell with a target cell (e.g., tumor cells, infected cells and others) [12,13]. Involved in the activation are more than 100 different genes whose products are localized on the cell surface, in the cytoplasm and nucleus (Figure 1). The final product of the cascade tumor necrosis factor (TNF) is released and additionally granzyme (GRZMB) and perforin (PRF1) are synthesized in cytotoxic granules and secreted by exocytosis to destroy the target cell directly (Figure 1) [12].

This study aimed to validate the protein expression of the NK cytotoxic signaling pathway identified by Lenk *et al.* [10] in AAA tissue. To accomplish this, we selected nine members, representing different stages of the activation, of the NK Cell Mediated Cytotoxicity Pathway and carried out histological analysis using tissues samples collected from AAA repair operations and infrarenal aortic samples from age-, sex- and ethnicity-matched controls. As NK cells are present in only low numbers in AAA based on previous studies [14,15], the cellular expression of these proteins in other inflammatory cells was characterized using markers for lymphatic cells (CD8+) and monocytes/macrophages (CD68+).

## 2. Results

### 2.1. Immunohistochemical Analysis Demonstrates Staining of Members of NK Cell Mediated Cytotoxicity Pathway in Human AAA Tissue

We studied the protein expression of nine different members of the NK Cell Mediated Cytotoxicity Pathway (VAV1, VAV3, PLCG1, PLCG2, HCST, TYROBP, PTK2B, TNFA, and GZMB; Figure 1) in human AAA and compared the results to aortic tissue samples taken from the infrarenal aortae of age- and sex-matched controls (Table 1). Eight (VAV1, VAV3, PLCG2, HCST, TYROBP, PTK2B, TNFA, and GZMB) of the corresponding mRNAs of these proteins had been shown to be significantly elevated and one (PLCG1) significantly decreased in AAA compared to non-aneurysmal aortae (Figure 1).

**Table 1:** Aortic tissue samples used for immunohistochemical staining.

Case ID	Age (Years)	Sex	Cause of Death	Classification
ME0101	53	Male	Gunshot Wound	Control
ME0105	53	Male	Unknown	Control
ME0205	78	Male	Cardiac Arrest	Control
ME0501	69	Female	Fall (Head Trauma)	Control
ME0503	54	Male	Cardiac Arrest	Control
ME0505	59	Female	Cardiovascular	Control
ME1001	88	Female	Trauma	Control
ME1003	44	Male	Overdose/Cardiovascular	Control
WSU052	70	Male	NA	AAA
WSU060	70	Male	NA	AAA
WSU068	72	Male	NA	AAA
WSU075	67	Male	NA	AAA
WSU080	64	Female	NA	AAA
WSU081	69	Male	NA	AAA

Tissue samples were obtained at autopsy or at operation, and were taken from the infrarenal abdominal aorta. All individuals were Caucasian. Tissue samples from WSU080 were used for both microarray expression study [10] and the current immunostaining study. The same autopsy samples have been used in our previous studies and have shown comparable performance in mRNA and protein analyses to samples taken from AAA operations [16,17,18,19,20]. NA, not applicable, since the sample was obtained during an AAA repair operation.

**Figure 2:**
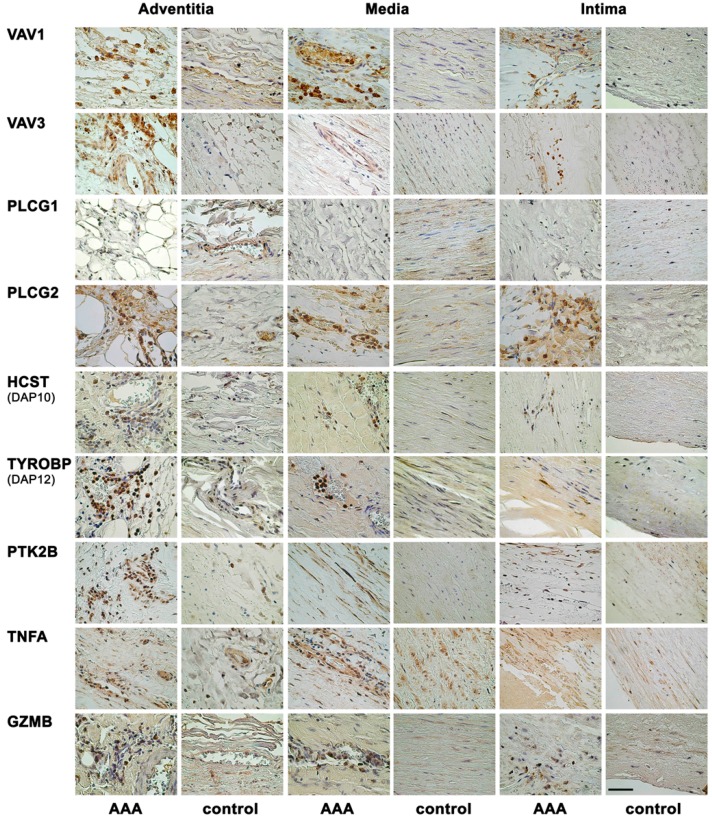
Immunohistochemical staining with antibodies against nine different proteins representing the NK pathway. Each row shows representative immunohistological images for the indicated antibodies on AAA tissue and control abdominal aortic tissue. Most antibodies demonstrated staining on inflammatory cells in the AAA tissue. See Table 1 and Table 2 for details on the aortic tissues and antibodies used, respectively. Scale bar = 50 μm.

Immunohistochemical results with representative images of the single staining are summarized in Figure 2. The antibody against VAV1 showed strong staining in AAA tissue, but no staining in control aorta. The staining was cytoplasmic and seen mainly in inflammatory cells in all layers of AAA wall. The positively stained inflammatory cells were likely granulocytes based on their large size and the lobed nuclei. Similarly, the antibody against VAV3 stained inflammatory cells in the adventitia, media and intima in AAA tissues, but gave no staining in the control aorta.

Consistent with mRNA studies (Figure 1), AAA tissue showed no staining against PLCG1 antibody, while control tissue had ubiquitous staining in endothelial cells of the vasa vasorum and in the vascular smooth muscle cells (VSMCs) (Figure 2). The other subunit of the phospholipase C, gamma protein, PLCG2, showed stronger staining in AAA than in control aortae. Inflammatory cells including lymphocytes and granulocytes (based on nuclear morphology) were PLCG2-positive. Additionally, in the AAA tissue, endothelial cells in the vasa vasorum of the adventitia, neovessels in the media and adipocytes in the adventitia showed strong staining for PLCG2.

Inflammatory cells (granulocytes and some lymphocytes) in AAA samples were also positive for HCST (DAP10) with no staining in control aortae (Figure 2). Antibody against TYROBP (DAP12) showed intense cytoplasmic and nuclear staining of inflammatory cells (lymphocytes and granulocytes) in adventitia and media of AAA tissue. Similarly, inflammatory cells (lymphocytes and granulocytes) had a strong staining against PTK2B (PYK2) in AAA tissue. Additionally, VSMCs showed cytoplasmic staining for PTK2B in AAA, but there was no staining in the control aortae (Figure 2).

The antibody against TNFA showed staining in AAA tissue mainly in adventitia and media (Figure 2). Inflammatory cells as well as endothelial cells of the vasa vasorum and the neovessels and VSMCs in the media were positive for TNFA. In control aortae, the few visible inflammatory cells and VSMCs showed a positive staining for TNFA. Staining for GZMB was present in inflammatory cells in all layers of AAA tissue, but control aortae were negative (Figure 2).

### 2.2. Immunohistochemical Analyses with Double Staining Show Co-Localization of Proteins Participating in the Early and Late Steps of the NK Pathway Activation

**Figure 3:**
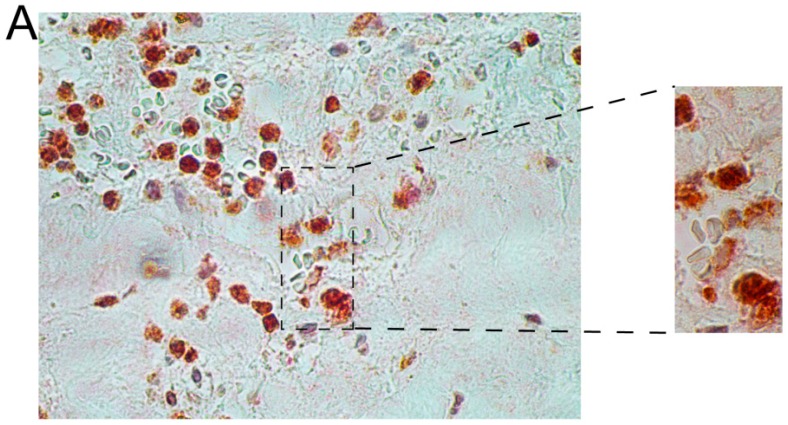

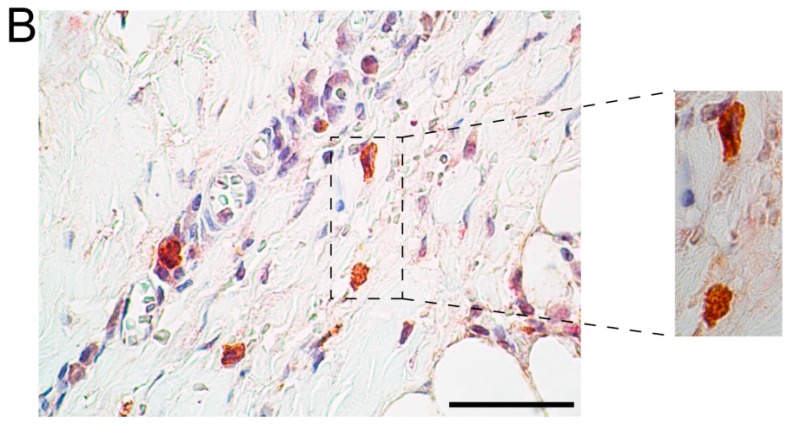
Double-staining with antibodies against HCST (red) and GMZB (brown) in AAA tissue. The donors were WSU060 (**A**); and WSU052 (**B**) for the AAA tissues used in the staining shown in the upper and lower panels, respectively. See Table 1 and Table 2 for details on the aortic tissues and antibodies used, respectively, and Supplementary Figure S1 for additional images. Scale bar = 50 µm.

**Figure 4:**
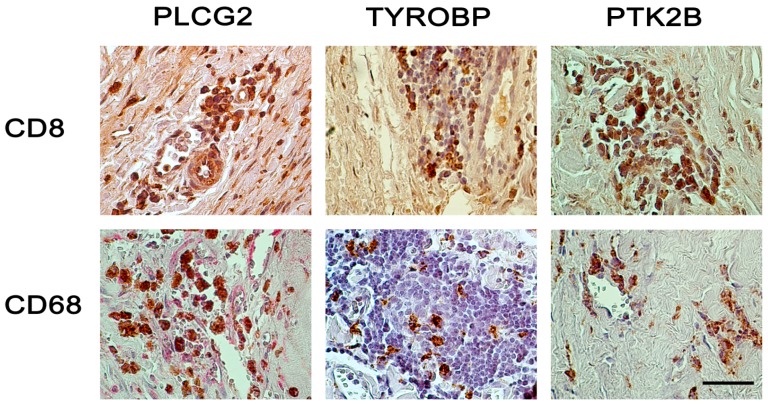
Double-staining with different combinations of antibodies in AAA tissue. CD68 staining identifies monocytes and macrophages, and CD8 identifies cytotoxic T-cells. For the CD8–PLCG2 double-staining (upper left image), brown color is for PLCG2 and red for CD8. For all other images, brown color is for CD8 or CD68 and red color is for PLCG2, TYROBP, or PTK2B. See Table 1 and Table 2 for details on the aortic tissues and antibodies used, respectively, and Supplementary Figures S2 and S3 for additional images. The slides were counterstained with hematoxylin, and nuclei stain blue-purple color. Lymphocytes show intense blue staining, since they have little cytoplasm. Scale bar = 50 µm.

Immunohistochemical results with representative images of the double staining are shown in Figure 3 and Figure 4. We first used double-staining to study the co-expression of two different members of the NK pathway. These studies revealed that inflammatory cells in AAA wall were positive for both HCST (DAP10) and GRZB (Figure 3 and Supplementary Figure S1). These findings indicated that HCST (DAP10) and GZMB, both of which are enzymes and act at different steps in the NK-pathway, were actively transcribed and translated into proteins in the same cells in the AAA tissue (Figure 3 and Supplementary Figure S1).

We also used two cell-specific markers, CD68 for macrophages/monocytes and CD8 for lymphocytes, to carry out double-staining experiments together with members of the NK pathway. Many inflammatory cells, mainly in the adventitia and media in the AAA wall stained positive for PLCG2, but only a few of those cells expressed CD8 (Figure 4 and Supplementary Figure S2). While nearly half of the inflammatory cells were CD8-positive lymphocytes, only a few of them also expressed TYROBP (Figure 4 and Supplementary Figure S2). CD8-positive leukocytes stained with antibody against PTK2B. There were, however, also CD8-negative cells which showed staining for PTK2B. This finding suggests that different cell types express PTK2B (Figure 4 and Supplementary Figure S2). 

Double staining was seen with anti-CD68 and anti-TYROBP indicating that macrophages express TYROBP (Figure 4 and Supplementary Figure S3). CD68-positive macrophages also showed cytoplasmic staining against anti-PLCG2. The double staining showed that PLCG2 had ubiquitous expression and staining for anti-PLCG2 was also seen in other inflammatory and epithelial cells (Figure 4 and Supplementary Figure S3). Macrophages (CD68-positive cells) were also PTK2B-positive (Figure 4 and Supplementary Figure S3), a finding consistent with previous studies showing expression of this gene in macrophages and demonstrating that it is required for macrophage migration and function [21,22].

## 3. Discussion

Based on histopathological examination, the wall of AAA exhibits features of chronic inflammatory disease and vascular inflammation [23]. In a recent review Libby and Hansson [24] emphasized the role inflammation and the immune system play in many arterial diseases including AAAs. They called it the “immunopathogenesis of arterial diseases” and urged to “think beyond the intima and include the other layers of the artery wall”. Previous studies have suggested that the innate immune system, such as the complement cascade [16], and the adaptive immune system play roles in AAA, and infiltrating leukocytes are present in AAA [23,25]. Several previous studies provided evidence that NK cytotoxic pathway contributes to AAA pathobiology even in the macroscopically non-inflammatory AAAs. These included studies that demonstrated the presence of NK cells and NKT cells in the aneurysmal wall [25], found large numbers of NK cells in the peripheral blood of AAA patients [23], and discovered increased mRNA expression of natural killer cell associated protein (NKTR) in the aortic neck [26]. The NK cells from patients with AAA were also shown to have increased cytotoxicity [23]. Additionally the higher percentage of NK cells and the higher cytotoxicity was persistent after AAA repair [23], which could be a sign of systemic disease. Furthermore, one study showed that AAA patients have increased serum levels of TNFA [27] and another study showed that T cells isolated from AAA patients secreted more TNFA *in vitro* [15]. All of these studies provided evidence for the NK pathway activation in AAA.

Previous results from a microarray-based mRNA expression study comparing AAA and non-aneurysmal infrarenal aortic tissue showed an increase in 43 of 49 differentially expressed genes from the NK pathway [10]. Similarly, prior work using the apolipoprotein E deficient mouse model of aortic aneurysms found an activation of the same pathway in the aortas of animals treated with angiotensin II as compared with controls [28]. Nine (*HCST*, *TYROBP*, *PTK2B*, *VAV1*, *RAC2*, *FCGR3*, *FCER1G*, *LCP2*, and *CD48*) of the 10 genes described as up-regulated in the mouse study were also up-regulated in our study of human AAA tissue [28]. The animal study did not, however, show an increase of the final products *TNF* and *GZMB* in the aneurysm group [28]. This is somewhat surprising since the complete activation of the NK pathway would require these gene products and they were clearly elevated in human AAA based on the current and previous studies [29]. TNFA functions in cell apoptosis and induces MMP expression [27]; GZMB can lyse target cells [12,30]. Perforin is also known to be expressed in NK cells and cytotoxic T-cells in human AAA [15,31].

In the current study, the immunostaining of selected members in the NK-pathway confirmed the mRNA expression results on the protein level (Figure 2). The histological investigation suggested NK cells were not the only cell type involved in the activation of the NK pathway because most inflammatory cells showed positive staining (Figure 2). A double staining with GZMB and HCST demonstrated that products of the beginning of the activation pathway and the final products were produced in the same cell, providing evidence for the complete activation of the pathway (Figure 3 and Supplementary Figure S1).

As the number of NK cells is reported to be small in AAA [14,15], it was of interest to see which other cell types have an activated NK pathway. The inflammation in the AAA wall is characterized mainly by CD3+ T cells and B cells, a few macrophages and NK cells [14,32]. Macrophages (CD68+) and cytotoxic T cells (CD8+) are thought to play an important role in AAA development by expressing perforin (PRF1) which can damage the membrane of the target cell [15]. The mRNA for *PRF1* was detectable only in AAA tissue, not in the non-aneurysmal aorta. Abundant GZMB expression in human AAA tissue by immunostaining was demonstrated in a previously published study [30]. In the apolipoprotein E mouse model, GZMB was shown to act independently of perforin in AAA and absence of GZMB decreased the rate of AAA formation [30]. These results were consistent with the microarray-based expression study [10].

TNFA is one of the best studied cytokines in AAA, and has been shown consistently to be upregulated in AAA [29]. Increased levels of TNFA in serum and aorta have been described in human AAA [29,33]. In a CaCl_2_-induced AAA mouse model, blockage of TNFA attenuated aneurysm formation [34]. In another study using cultured murine SMCs, TNF activated Mmp9 and Timp1 which are known to play important roles in the remodeling of the ECM [35]. The increased expression of TNFA in the RNA microarray study [10] and the increased protein levels found in the current immunostaining study add further evidence that TNFA is important in AAA pathogenesis.

Other components of the NK pathway such as VAV1, VAV3, PLCG1, PLCG2, HCST, TYROBP, and PTK2B have not been investigated in AAA previously. These proteins were investigated in several studies focusing on other vascular conditions including atherosclerosis, platelet activation and hyperlipidemia as well as activation of macrophages [36,37,38,39,40,41]: VAV proteins play an important role in foam cell (which are lipid-laden macrophages and the earliest form of atherosclerotic lesions) formation [39]. Another study showed that VAV1 and VAV3 play a role in increased platelet reactivity associated with hyperlipidemia [36]. Thus, it is possible that the increased expression of VAV3 in AAA tissue is due to intraluminal thrombus formation commonly seen in AAA. Interestingly, a mutation in the *PLCG2* gene led to increased activity of PLCG2 and a severe autoimmune disease [41]. Since AAA is also an autoimmune disease, one could speculate that the increased levels of PLCG2 seen in the current study contribute to AAA pathophysiology. TYROBP (DAP12) has also been studied in metabolic, cardiovascular and inflammatory diseases [38].

PLCG1 is necessary for the activation of VAV1 in macrophages and is a protein which plays a critical role in intracellular calcium signaling [39]. The calcium signaling pathway was also identified among enriched pathways in a recent microarray-based study on human AAA [17]. PTK2B (PYK2) is activated in regulation of mononuclear phagocytosis to atherosclerotic lesions [37]. A role for HCST (DAP10) in aneurysm or atherosclerosis has not been described previously, but the NKD2D/DAP10 receptor complex is known to play a crucial role in macrophage activation [40] further supporting the results of macrophage activation seen in the AAA tissue.

The results of the double immunostaining revealed the complexity of the inflammatory cells in the AAA wall. The expression of the components of the NK pathway is not restricted to one cell type. Double staining with antibodies against macrophages/monocytes (CD68) combined with antibodies against TYROBP, PLCG2, and PTK2B revealed that CD68+ cells were involved in the activation of the NK Cell Mediated Cytotoxicity Pathway. PLCG2 in particular had ubiquitous expression not restricted to inflammatory cells. Double staining with anti-CD8 as a marker for cytotoxic T cells showed that nearly 50% of the lymphatic cells in the AAA wall were CD8+, a number which is considerably higher than the 20%–30% described previously [31,32]. Yet, only few CD8+ cells expressed TYROBP, PLCG2 or PTK22B suggesting that there is a special subgroup of CD8+ cell type. PTK2B and PLCG1 were also expressed in other cell types. Clearly, further studies are needed to characterize the cell types positive for members of the NK pathway. It is of interest to note that the activation of the NK Cell Mediated Cytotoxicity Pathway is not restricted to the NK cells in human AAA tissue.

The NK Cell Mediated Cytotoxicity Pathway is known to have features of the innate immune and the adaptive immune systems [42] which could explain the expression of the members of the pathway in different cell types. In particular, the cytotoxic CD8+ T cell is a close relative of the NK cell [42]. An increase in cytotoxic cells including NK, NKT and CD8 T-cells and macrophages may contribute to AAA pathogenesis by the production of cytotoxic mediators resulting in SMC apoptosis.

Limitations of the study are the use of surgically removed human aneurysmal aortic tissue which shows already end-stage disease, so it is not clear if the NK Cell Mediated Cytotoxicity Pathway is already activated in the early stages of the disease. The study was restricted to the aortic tissue. It is, therefore, not possible to say if the changes seen constitute a systemic or local reaction.

## 4. Experimental Section

### 4.1. Human Aortic Tissue Samples

Aortic wall tissue specimens (six AAA patients; ages ranging from 64 to 72 years, mean age 68.67 ± 2.56 years) were collected from patients undergoing elective AAA repair operations at the Harper University Hospital, Detroit, MI, USA. Non-aneurysmal infrarenal aortic samples were collected at autopsies (*n* = 8; donor ages ranging from 44 to 88 years and mean of 62.25 ± 13.87 years). The same autopsy samples have been used in our previous studies and have shown comparable performance in mRNA and protein analyses to samples taken from AAA operations [16,17,18,19,20]. Samples were stored in phosphate-buffered formalin and embedded in paraffin. Donor information for all samples is listed in Table 1. The collection of the human tissues was approved by the Institutional Review Board of Wayne State University, Detroit, MI, USA.

### 4.2. Microarray-Based Gene Expression Studies

In our previous study [10], we used two microarray platforms to generate global mRNA expression profiles for both aneurysmal (*n* = 6) and non-aneurysmal (*n* = 7) human infrarenal abdominal aorta. The details on these studies have been described previously, and the microarray data can be obtained at the Gene Expression Omnibus (GEO) database (Series #GSE7084)) [43,44].

The results revealed 3274 differentially expressed genes between aneurysmal and control aortic tissue with False Discovery Rate (FDR) of <0.05. Analysis of biological pathways, including Gene Ontology (GO) and KEGG, indicated extreme overrepresentation of immune related categories. The enriched categories included the GO category Immune Response (GO: 0006955; FDR = 2.1 × 10^−14^), and the KEGG pathway NK Cell Mediated Cytotoxicity (hsa04650; FDR = 5.9 × 10^−6^). In the NK Cell Mediated Cytotoxicity pathway, 86/127 probed genes were expressed in either AAA or controls (AAA: 84; control: 75). Interestingly, 49/86 (57%) of the expressed genes in this pathway were significantly differentially expressed between AAA and the controls (Figure 1; see also the Additional File 4 in Lenk *et al.* [10]). There were 43 genes with increased mRNA levels in AAA whereas only six genes (*MICA*, *PTPN11*, *SHC4*, *PLCG1*, *PIK3R1*, and *RAC1*) had decreased mRNA levels in AAA (Figure 1). There were also 12 genes with detectable expression in only the AAA tissue (*GZMB*, *CD244*, *FYN*, *ITGAL*, *KIR3DL1*, *KLRK1*, *NCR3*, *PRF1*, *SH2D1B*, *SH3BP2*, *TNF*, *ZAP70*), and two genes (*PIK3R3*, *NFATC4*) expressed only in control aortic tissue (Figure 1).

Gene symbols available from the National Center for Biotechnology Information (NCBI) [45] were used.

### 4.3. Immunohistochemical Analysis

Immunostaining was carried out with formalin-fixed paraffin-embedded tissue sections as described previously [46]. The slides were incubated with a primary antibody (Table 2) on an automatic immunostainer (Dako Autostainer, Carpinteria, CA, USA). A secondary antibody with peroxidase labelled polymer conjugated to either goat anti-mouse or goat anti-rabbit immunoglobulins (Dako, Glostrup, Denmark) was used and the signal was detected with substrate chromogen solution (Dako). Antibodies for each protein were first tested on tissue known to contain the protein of interest as positive controls. Non-specific IgG antibody in lieu of primary antibody served as a negative control and gave no staining. Four of the selected antibodies (CD244, SH2D1A; RAC1 and RAF1; Table 2) showed no staining even with tissues used as positive controls. In addition, the antibody against LAT showed a good specific staining in the positive controls, but no staining in AAA or control aorta.

Double staining was performed with either an antibody against CD68 (a marker for the macrophages/monocytes) or an antibody against CD8A (a marker for cytotoxic T-cells), and the antibodies against TYROBP (DAP12), PTK2B (PYK2), or PLCG1. Double staining with antibodies against HCST (DAP10) and GMZB was also carried out. The experiments were carried out according to the manufacturer’s instructions using kit EnVision™ G/2 Doublestain System, Rabbit/Mouse DAB+/Permanent Red (K5261; Dako).

For evaluation of the stained slides microscope Nikon OPTIPHOT-2 (Tokyo, Japan) and Nikon Digital Camera DS-Fi2 were used. The camera software was NIS-Elements V4.10 (Nikon). Images were assembled for figures using Image J software (National Institutes of Health, Bethesda, MD, USA) [47].

**Table 2:** Primary antibodies used for immunohistochemical staining of aortic tissue samples.

Gene Symbol *	Gene ID *	Protein Symbols	Full Name	Catalog Number	Supplier	Species
*PTK2B*	2185	PTK2B, PYK2, PKB, PTK	Protein tyrosine kinase 2 β	ab55358 ^¶^	Abcam, Cambridge, MA, USA	Rabbit polyclonal
*LAT*	27040	LAT, pp36	Linker for activation of T cells	ab32070 ^§^	Abcam, Cambridge, MA, USA	Rabbit monoclonal
*VAV3*	10451	VAV3	Vav 3 guanine nucleotide exchange factor	ab40889	Abcam, Cambridge, MA, USA	Rabbit polyclonal
*RAC2*	5880	RAC2, HSPC022, p21-Rac2, EN-7	Ras-related C3 botulinum toxin substrate 2 (rho family, small GTP binding protein Rac2)	ab2244 ^†^	Abcam, Cambridge, MA, USA	Goat polyclonal
*TNF*	7124	TNF, TNFA, TNFSF2, DIF	Tumor necrosis factor	ab6671	Abcam, Cambridge, MA, USA	Rabbit polyclonal
*CD244*	51744	CD244, 2B4, NAIL, NKR2B4, SLAMF4	CD244 molecule, natural killer cell receptor 2B4	HPA010628 ^†^	Sigma-Aldrich, St. Louis, MO, USA	Rabbit polyclonal
*TYROBP*	7305	DAP12, KARAP, PLOSL	TYRO protein tyrosine kinase binding protein	sc-20783 ^¶^	Santa Cruz Biotechnology Inc., Santa Cruz, CA, USA	Rabbit polyclonal
*HCST*	10870	DAP10, KAP10, PIK3AP	Hematopoietic cell signal transducer	sc-10531 ^¶^	Santa Cruz Biotechnology Inc., Santa Cruz, CA, USA	Goat polyclonal
*SH2D1A*	4068	SAP, LYP, DSHP, MTCP1, EBVS	SH2 domain containing 1A	sc-8333 ^†^	Santa Cruz Biotechnology Inc., Santa Cruz, CA, USA	Rabbit polyclonal
*VAV1*	7409	VAV1, VAV	Vav 1 guanine nucleotide exchange factor	sc-132	Santa Cruz Biotechnology Inc., Santa Cruz, CA, USA	Rabbit polyclonal
*PLCG1*	5335	PLCG1, PLC1, NCKAP3, PLC148	Phospholipase C, gamma 1	sc-7290	Santa Cruz Biotechnology Inc., Santa Cruz, CA, USA	Mouse monoclonal
*PLCG2*	5336	PLCG2, FCAS3, APLAID	Phospholipase C, gamma 2 (phosphatidylinositol-specific)	sc-5283 ^¶^	Santa Cruz Biotechnology Inc., Santa Cruz, CA, USA	Mouse monoclonal
*RAC1*	5879	RAC1, MIG5, TC-25, p21-Rac1	Ras-related C3 botulinum toxin substrate 1 (rho family, small GTP binding protein Rac1)	05-389 ^†^	Millipore Corporation, Billerica, MA, USA	Mouse monoclonal
*RAF1*	5894	RAF1, NS5, CRAF	v-raf-1 murine leukemia viral oncogene homolog 1	sc-7267 ^†^	Santa Cruz Biotechnology Inc., Santa Cruz, CA, USA	Mouse monoclonal
*GZMB*	3002	GZMB, HLP, CCPI, CGL1, SECT, CTLA1, CTSGL1	Granzyme B (granzyme 2, cytotoxic *T*-lymphocyte-associated serine esterase 1)	sc-1969 ^¶^	Santa Cruz Biotechnology Inc., Santa Cruz, CA, USA	Goat polyclonal
*CD8A*	925	CD8, MAL, p32, Leu2	CD8a molecule	M7103 ^¶^	Dako, Glostrup, Denmark	Mouse monoclonal
*CD68*	968	CD68, GP110, LAMP4, SCARD1	CD68 molecule	M0876 ^¶^	Dako, Glostrup, Denmark	Mouse monoclonal

* HGNC approved gene and IDs are available from the HUGO Gene Nomenclature Committee [48] or National Center for Biotechnology Information [45]; ^†^ Antibody did not stain positive controls and was not used for staining of aortic samples; ^§^ Antibody stained positive controls but did not stain aortic tissue samples and results are, therefore, not reported here; ^¶^ Used for double-staining.

## 5. Conclusions

The NK Cell Mediated Cytotoxicity Pathway is activated in human AAA tissue based on both mRNA expression data and protein analysis using immunostaining. We demonstrated that components of the NK pathway and TNFA, GZMB and PRF1, the highly destructive end products, are produced not only in NK cells but also in cytotoxic T cells (CD8+) and macrophages (CD68+) in the aneurysmal aortic wall. The exact role for inflammation in AAA pathogenesis is still an unanswered question; on one hand, there is evidence that inflammation could contribute to AAA progression and on the other hand, inflammation could result from an injury in the aortic wall.

## Supplementary Materials

Supplementary materials can be found at http://www.mdpi.com/1422-0067/16/05/11196/s1.
