# Supplementary material for: Immunohistochemical Analysis of the Natural Killer Cell Cytotoxicity Pathway in Human Abdominal Aortic Aneurysms
Source: Int J Mol Sci. 2015 May 18;16(5):11196–212. doi: 10.3390/ijms160511196 (PMC4463696; doi:10.3390/ijms160511196)
Supplement: Supplementary file 1 [file ijms-16-11196-s001.pdf]

Supplementary Information

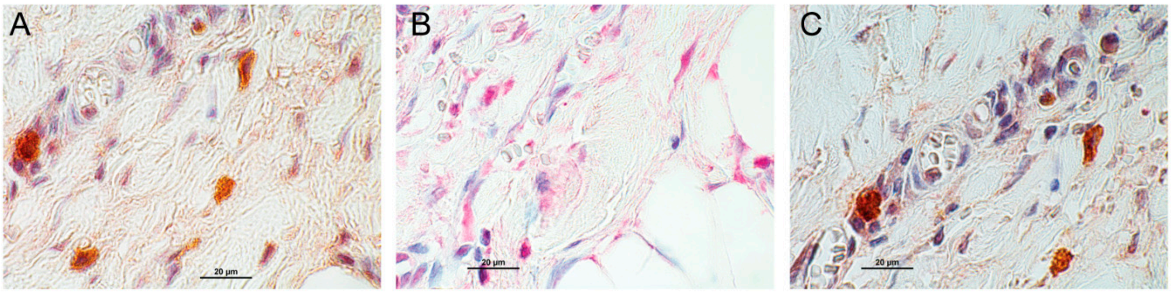

Figure S1. Co-expression of GRZ and HCST in AAA tissue. Single staining of GRZ (**A**); and HCST (**B**); as well as double staining (**C**) of GRZ and HCST are shown in the same tissue sample (WSU052) using serial sections. Macrophages show simultaneous staining for GRZ (brown) and HCST (red). Scale bar = 20 µm.

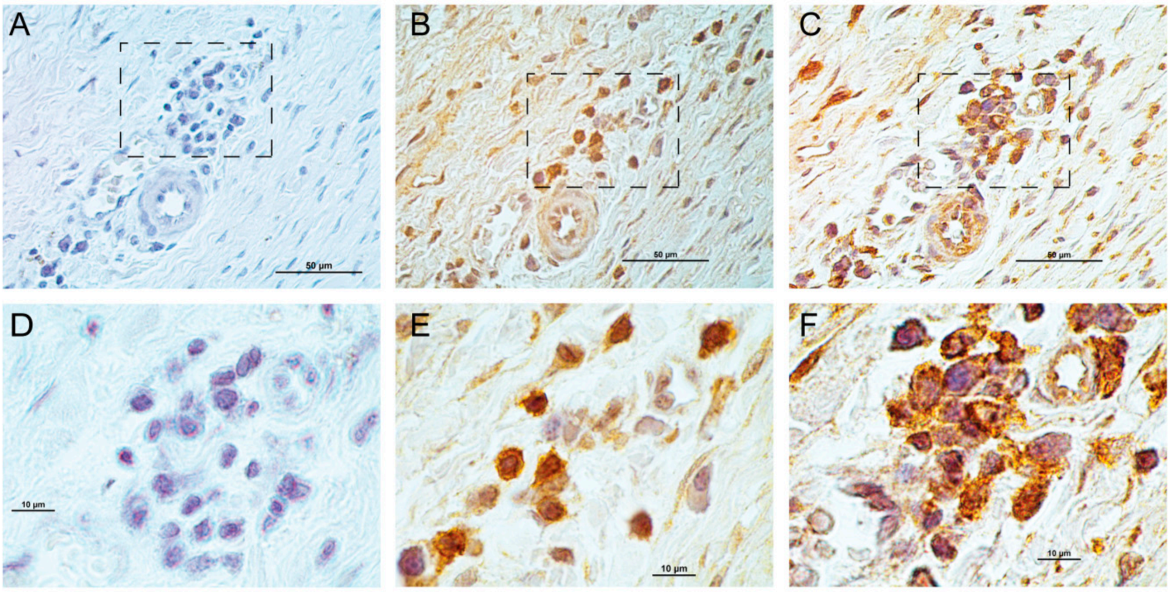

Figure S2. Single staining of aortic AAA tissue with an antibody against CD8 (**A**,**D**); and double staining for CD8-TYROBP (**B**,**E**); and CD8-PLCG2 (**C**,**F**). The single staining with anti-CD8 shows red staining in the cytoplasm of several lymphocytes, which can be seen better if the image D is viewed at 400%. The double staining images for CD8 (brown) and TYROBP (red) in images B and E, as well as for CD8 (red) and PLCG2 (brown) in images C and F demonstrate that CD8 positive lymphocytes express TYROBP and PLCG2. Lower magnification images are on the top row (**A**–**C**) and higher magnification images of the areas boxed with dashed lines are shown in the lower row (**D**–**F**). The images were taken from the same tissue sample (WSU075) using serial sections. The slides were counterstained with hematoxylin, and nuclei stain blue-purple color. Lymphocytes show intense blue staining, since they have little cytoplasm. Scale bar = 50 µm in the upper row and 10 µm in the lower row.

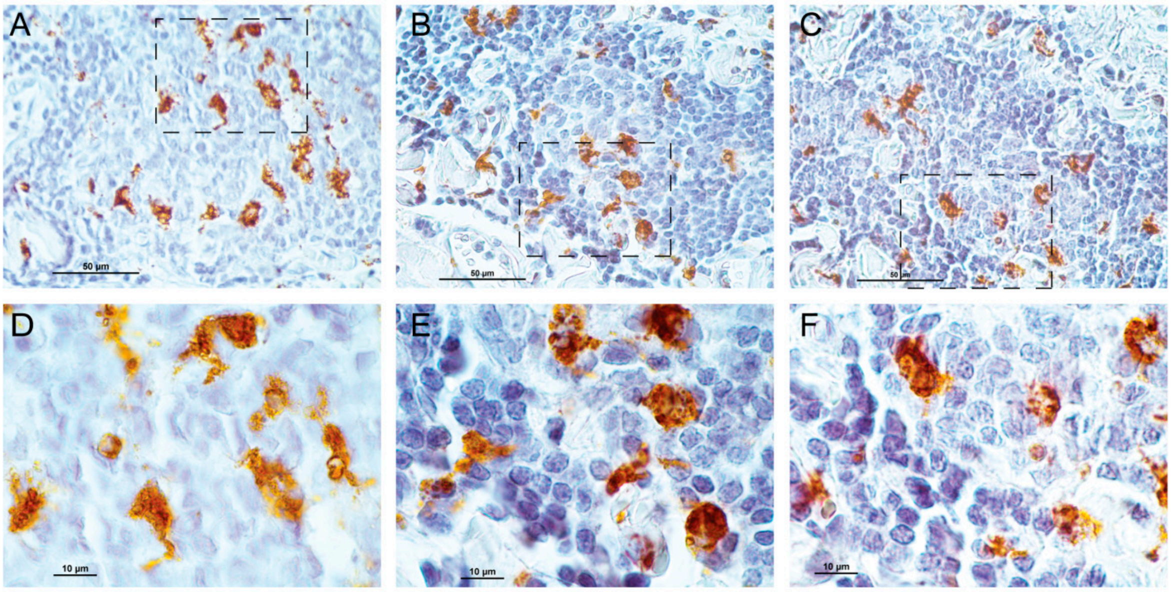

Figure S3. Single staining of aortic AAA tissue with an antibody against CD68 (**A**,**D**); and double staining for CD68-TYROBP (**B**,**E**); and CD68-PTK2B (**C**,**F**). Intense brown staining is seen in monocytes/macrophages (**A**,**D**). The double staining for CD68 (brown) and TYROBP (red) in the images B and E, and CD68 (brown) and anti-PLCG2 (red) in images (**C**) and (**F**) shows that monocytes/macrophages express TYROBP and PLCG2. Lower magnification images are on the top row (**A**–**C**) and higher magnification images of the areas boxed with dashed lines are shown in the lower row (**D**–**F**). The images were taken from the same tissue sample (WSU052) using serial sections. The slides were counterstained with hematoxylin, and nuclei stain blue-purple color. Lymphocytes show intense blue staining, since they have little cytoplasm. Scale bar = 50 µm in the upper row and 10 µm in the lower row.
